# Dynamic Modeling of Indole Glucosinolate Hydrolysis and Its Impact on Auxin Signaling

**DOI:** 10.3389/fpls.2018.00550

**Published:** 2018-04-26

**Authors:** Daniel Vik, Namiko Mitarai, Nikolai Wulff, Barbara A. Halkier, Meike Burow

**Affiliations:** ^1^DynaMo Center, Copenhagen Plant Science Centre, Department of Plant and Environmental Sciences, University of Copenhagen, Frederiksberg, Denmark; ^2^Center for Models of Life, Niels Bohr Institute, University of Copenhagen, Copenhagen, Denmark

**Keywords:** mathematical modeling, indole glucosinolate hydrolysis, auxin signaling, myrosinases, specifier protein, nitrilase, auxin antagonist

## Abstract

Plants release chemicals to deter attackers. *Arabidopsis thaliana* relies on multiple defense compounds, including indol-3-ylmethyl glucosinolate (I3G), which upon hydrolysis initiated by myrosinase enzymes releases a multitude of bioactive compounds, among others, indole-3-acetonitrile and indole-3-acetoisothiocyanate. The highly unstable isothiocyanate rapidly reacts with other molecules. One of the products, indole-3-carbinol, was reported to inhibit auxin signaling through binding to the TIR1 auxin receptor. On the contrary, the nitrile product of I3G hydrolysis can be converted by nitrilase enzymes to form the primary auxin molecule, indole-3-acetic acid, which activates TIR1. This suggests that auxin signaling is subject to both antagonistic and protagonistic effects of I3G hydrolysis upon attack. We hypothesize that I3G hydrolysis and auxin signaling form an incoherent feedforward loop and we build a mathematical model to examine the regulatory network dynamics. We use molecular docking to investigate the possible antagonistic properties of different I3G hydrolysis products by competitive binding to the TIR1 receptor. Our simulations reveal an uncoupling of auxin concentration and signaling, and we determine that enzyme activity and antagonist binding affinity are key parameters for this uncoupling. The molecular docking predicts that several I3G hydrolysis products strongly antagonize auxin signaling. By comparing a tissue disrupting attack – e.g., by chewing insects or necrotrophic pathogens that causes rapid release of I3G hydrolysis products – to sustained cell-autonomous I3G hydrolysis, e.g., upon infection by biotrophic pathogens, we find that each scenario gives rise to distinct auxin signaling dynamics. This suggests that plants have different defense versus growth strategies depending on the nature of the attack.

## Introduction

**FIGURE 1:**
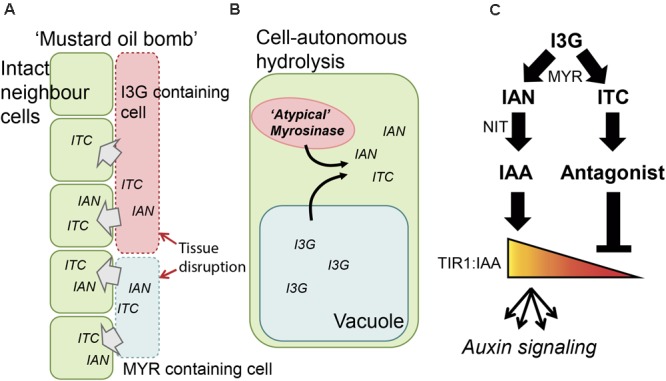
Schematic representation of I3G hydrolysis and auxin signaling. **(A)** Triggering of the ‘mustard oil bomb.’ MYR and I3G are compartmentalized in myrosin and S-cells, respectively. Upon attack the cells are disrupted and MYR and I3G get into contact forming IAN and ITC in the vicinity of intact neighboring cells. **(B)** Cell-autonomous I3G hydrolysis in an intact cell. I3G and atypical myrosinases are kept in separate intracellular compartments, but can be, upon infection with biotropic pathogens, translocated and brought into contact, releasing IAN and ITC intracellularly. **(C)** Outline of the regulatory network. I3G is hydrolyzed by MYR and then converted into IAN and ITC. IAN is further converted into IAA by NIT, and IAA binds TIR1 to elicit auxin signaling. ITC is non-enzymatically converted to compounds that display antagonistic behavior toward TIR1:IAA complex formation (e.g., I3C).

Plants have evolved numerous chemicals to defend themselves against attack from herbivores and pathogens. These are fascinating examples of complex biological designs. A well-studied example is the two-component ‘mustard oil bomb’ characteristic of cruciferous plants (**Figure 1A**) (Lüthy and Matile, 1984; Halkier and Gershenzon, 2006; Wittstock and Burow, 2010). The ‘mustard oil bomb’ consists of the sulfur-rich GLS and thioglucosidase enzymes referred to as MYRs. Hydrolysis of GLS by MYR leads to GLS aglucones that can spontaneously rearrange into isothiocyanates (ITCs) – reactive compounds with antimicrobial and insecticidal activities (Wittstock et al., 2003; Wittstock and Burow, 2010). In the presence of NSPs, hydrolysis does not lead to ITC formation, but instead gives nitriles or other GLS hydrolysis products (Burow et al., 2006, 2009; Wittstock and Burow, 2010; Wittstock et al., 2016). Thus, GLS and their hydrolysis products give rise to complex mixtures of products with diverse functions associated with environmental interactions (Beekwilder et al., 2008; de Vos et al., 2008; Agerbirk et al., 2009; Burow et al., 2009; Wittstock and Burow, 2010).

The two main components, GLS and MYR, are spatially separated in the plant. This can be in separate cells, i.e., the GLS-containing S-cells and the MYR-containing myrosin cells (Andréasson et al., 2001; Koroleva et al., 2010), or in separate intracellular compartments (Grob and Matile, 1979; Andréasson et al., 2001). Upon tissue breakage – e.g., by attack by chewing insect or necrotrophic pathogen infection – the boundaries are broken and GLS are hydrolyzed by MYR (Bones and Iversen, 1985; Andréasson et al., 2001) (**Figure 1A**). Upon infection with biotrophic pathogens, the plant responds with cell-autonomous hydrolysis (i.e., independent of tissue breakage) of tryptophan-derived indole GLS by atypical MYRs (e.g., PEN2 and PYK10) (**Figure 1B**) (Bednarek et al., 2009; Clay et al., 2009; Nakano et al., 2014, 2017; Zhao et al., 2015). The effects elicited by indole GLS hydrolysis products go beyond toxicity and range from mechanistic protection – such as increased callose deposition to strengthening of the cell wall and inhibition of cell penetration (Clay et al., 2009) – to strategies such as modulation of the hypersensitive response (Zhao et al., 2015).

Hydrolysis of I3M GLS (I3G) is a possible source of IAA, the primary plant auxin. The nitrile hydrolysis product, IAN, is a known *in vitro* substrate for NIT capable of converting IAN into IAA (Normanly et al., 1997; Vorwerk et al., 2001; Janowitz et al., 2009; Fu et al., 2016). Auxins are phytohormones generally associated with plant growth, e.g., by weakening of cell wall (Fu and Wang, 2011). It is believed that auxins attenuate plant defense by promoting growth over defense (Naseem et al., 2015), as increased auxin inhibits biosynthesis of salicylic acid – the major pathogen-induced defense hormone (Wang et al., 2007; Robert-Seilaniantz et al., 2011). Certain pathogens exploit this and actively synthesize and secrete auxins, possibly to facilitate successful infection (Yamada, 1993; Fu and Wang, 2011; Huot et al., 2014). Whether increased levels of auxin upon fungal infection of plant tissues originate from the pathogen or the plant is, however, not always clear. While auxins do not seem to be strictly required for pathogenicity (Chanclud and Morel, 2016), they may still play a critical role in fine-tuning plant–pathogen interactions.

The highly reactive ITC hydrolysis product gives rise to a multitude of different compounds (Agerbirk et al., 2009). One of these, I3C, was recently reported to exhibit auxin-antagonistic behavior via its competitive binding to TIR1 – the major auxin receptor (Katz et al., 2015a,b). This proposes a function of an I3G hydrolysis product as inhibitor of auxin signaling upon attack. Breakdown of specifically I3G – and not the modified indole GLS, such as 4-methoxy-indol-3-ylmethyl GLS – thus represent a molecular link between plant defense and growth. Thus, in addition to exerting its direct defense function, I3G can impact auxin signaling through both a positive and a negative route, thereby constituting a feedforward loop in a regulatory network. More specifically, this suggests that I3G breakdown and auxin signaling form a ‘type 3’ incoherent feedforward loop which enables pulse-like behavior and conditional regulation (**Figure 1C**) (Mangan and Alon, 2003; Alon, 2007; Csikász-Nagy et al., 2009; Tyson and Novák, 2010; Zhang et al., 2011; Semsey, 2014).

Physiological changes are ultimately the result of an organism’s ability to respond to external and internal signals. Regulatory networks are essential for information processing and decision making. Because of this, we need to understand the properties of the underlying regulatory network if we wish to gain insights into physiological responses. In cruciferous plants, the I3G-auxin loop may be part of the regulatory network balancing growth and defense strategies in response to attack as external signal. Here, we examined the dynamic properties of a possible regulatory network of I3G hydrolysis and auxin signaling. We propose a regulatory network consisting of a negative regulator through ITC-derived compounds and a positive enforcement through the NSP-directed production of the IAA precursor, IAN. We furthermore build the corresponding mathematical model and simulate the outcomes of I3G hydrolysis on auxin signaling (monitored as TIR1:IAA complex formation) using two scenarios: triggering of the ‘mustard oil bomb’ and sustained cell-autonomous hydrolysis. By bringing together previous experimental findings into a mathematical model and examining the proposed regulatory network, we investigate the effects of I3G hydrolysis on the dynamics of auxin signaling. Our simulations suggest that several of the I3G hydrolysis products may antagonize auxin signaling via competitive binding to the TIR1 receptor. We find that the two scenarios display different dynamics. Triggering of the ‘mustard oil bomb’ generates a pulse, which potentially serves as a signal being propagated to the surrounding cells. Sustained cell-autonomous hydrolysis, however, would enable a long-term uncoupling of auxin concentration and auxin signaling, which could play a role in auxin homeostasis under pathogen infection.

## Methods

### Mathematical Modeling

A series of ordinary differential equations were used to model the ‘incoherent feedforward loop’ of I3G breakdown and its effect on TIR1:IAA complex formation:

(1) $$\begin{array}{c} \frac{dIAN}{dt}=\;\beta *MYR*\alpha *\frac{V_{max,MYR}*I3G}{K_{M,MYR}+I3G}\;\;-\tau_{IAN}*IAN \end{array}$$

(2) $$\begin{array}{c} \frac{dAnt}{dt}\;=\left(1-\beta \right)*MYR*\alpha *\frac{V_{max,MYR}*I3G}{K_{M,MYR\;}+\;I3G}-\tau_{Ant}*Ant-k_{on,TIR1:Ant}*\left(TIR1-TIR1:IAA-TIR1:Ant\right)*\;Ant+k_{off,TIR1:Ant}*TIR1:Ant \end{array}$$

(3) $$\begin{array}{c} \frac{dIAA}{dt}\;=\theta +NIT*\frac{V_{max,NIT}*IAN}{K_{M,NIT\;}+IAN}-\tau_{IAA}*IAA-k_{on,TIR1:IAA}*\left(TIR1-TIR1:IAA-TIR1:Ant\right)*\;IAA*+k_{off,\;TIR1:IAA}*TIR1:IAA \end{array}$$

(4) $$\begin{array}{c} \frac{dTIR1:IAA}{dt}\;=k_{on,TIR1:IAA}*\;\left(TIR1-TIR1:IAA-TIR1:Ant\right)*\;IAA-k_{off,\;TIR1:IAA}*TIR1:IAA \end{array}$$

(5) $$\begin{array}{c} \frac{dTIR1:Ant}{dt}\;=k_{on,TIR1:Ant}*\;\left(TIR1-TIR1:IAA-TIR1:Ant\right)*\;Ant-k_{off,\;TIR1:Ant}*TIR1:Ant \end{array}$$

Where β is a value ranging from 0 to 1 representing the influence of NSP on MYR-catalyzed production of IAN. The α parameter is a Boolean value enabling I3G hydrolysis. The degradation rates of the various compounds are written with τ. Synthesis of IAA from other sources than I3G breakdown is written by 𝜃. Enzyme kinetic parameters for maximum velocity and Michaelis–Menten constant are denoted by *V*_max_ and *K*_M_, respectively. Binding rates *k_on_* and *k_off_* are derived from the dissociation constants *K*_D_ (*K*_D_ = *k*_off_/*k*_on_) estimated from molecular docking. The dynamic variables of the model are: IAN, IAA, antagonist (an umbrella term for all ITC-derived hydrolysis products capable of binding TIR1), TIR1, TIR1:IAA and TIR1:antagonist.

Several assumptions were made to model this system: (1) translocation of molecules occurs instantaneously and is not prohibited by either diffusion or transport; (2) binding on-rates of both IAA and antagonist to TIR1 are instantaneous (i.e., 1 μM^-1^s^-1^ which is considerably faster than our timescale of minutes); (3) protein abundance (MYR, NIT, and TIR1) remains constant within simulated timescale; (4) IAA exists only as free IAA, and not in sequestered or conjugated forms; (5) the degradation rate of IAN (τ_IAN_) is unknown and thus arbitrarily set to be 0.1 min^-1^, which is comparable to τ_Ant_; (6) Synthesis of IAA from sources other than I3G hydrolysis (i.e., 𝜃) remains constant throughout simulated timescale with a steady-state IAA concentration of ∼0.02 μM (as estimated below); (7) MYR enzyme kinetics acquired using the structurally different GLS sinigrin as substrate also apply to I3G; (8) Chemical conversion of ITC to conjugates (or I3C) occur instantaneously, and is a complete conversion of all ITC molecules.

### Parameter Estimation

In addition to the enzyme kinetic values, our model requires several parameters derived from experimental results. The total amount of I3G has been determined experimentally to be ∼0.1 nmol in a 5-day-old Arabidopsis seedling of the Columbia-0 accession and the weight of a seedling at that stage was determined to be roughly 1 mg (not shown). Assuming that the density of a seedling is ∼1 g/mL (as most organic matter), a 5-day-old seedling occupies a volume of ∼1 μL, with a I3G concentration of ∼100 μM. The amount of primary auxin, IAA, has been reported to be between 2.5 and 5 pg/mg fresh weight in 5-day-old seedlings (Bhalerao et al., 2002), which corresponds to an IAA concentration of ∼0.02 μM. Querying the large pep2pro database of quantitative proteomic data we extracted relative abundances of NITs (At3g44310, At3g44300, and At3g44320), MYRs (At5g26000, At1g47600, At1g51470, At2g44490, and At3g09260) and TIR1 (At3g62980) in Arabidopsis root tissue (Baerenfaller et al., 2011; Hirsch-Hoffmann et al., 2012). Taking advantage of an absolutely quantified sucrose synthetase [At3g43190, 2.25 fmol/mg FW (Wienkoop et al., 2010)] for normalization, we acquired rough estimates of protein abundance (**Table 1**).

**Table 1:** Parameter list.

Parameters	Description	Value	Unit	Reference
I3G	Concentration of indole-3-methyl glucosinolate	100	μM^∗^	Vik et al., Unpublished results
MYR	TGG1 Concentration of myrosinase enzyme	0.0022	mg enzyme/L	pep2pro database (root tissue)
	TGG4	0.37	mg enzyme/L^∗^	–
	TGG5	0.26	mg enzyme/L	–
	PEN2	0.12	mg enzyme/L	–
	PYK10	25.53	mg enzyme/L	–
*V*_MAX,MY R_	TGG1 Maximum reaction velocity	2.3	μmol/min/mg enzyme	Andersson et al., 2009
	TGG4	12.2	μmol/min/mg enzyme^∗^	–
	TGG5	48.1	μmol/min/mg enzyme	–
	PEN2	7.50	μmol/min/mg enzyme	Bednarek et al., 2009
	PYK10	0.00063	μmol/min/mg enzyme	Nakano et al., 2017
*K*_M,MY R_	TGG1 Michaelis–Menten constant	45	μM	Andersson et al., 2009
	TGG4	245	μM^∗^	–
	TGG5	547	μM	–
	PEN2	722	μM	Bednarek et al., 2009
	PYK10	82	μM	Nakano et al., 2017
β	Influence of nitrile-specifiers proteins on myrosinases	0.8	^∗^	Wentzell and Kliebenstein, 2008
NIT	NIT1 Concentration of nitrilase enzyme	0.67	mg enzyme/L^∗^	pep2pro database (root tissue)
	NIT2	0.62	mg enzyme/L	–
	NIT3	0.20	mg enzyme/L	–
*V*_MAX,NIT_	NIT1 Maximum reaction velocity	0.038	μmol/min/mg enzyme^∗^	Vorwerk et al., 2001
	NIT2	0.018	μmol/min/mg enzyme	–
	NIT3	0.015	μmol/min/mg enzyme	–
*K*_M,NIT_	NIT1 Michaelis–Menten constant	11100	μM^∗^	Vorwerk et al., 2001
	NIT2	7400	μM	–
	NIT3	30100	μM	–
TIR1	Total concentration of TIR1 receptor protein	1.595^∗^10^-5^	μM^∗^	pep2pro database (root tissue)
*K*_D,TIR1:IAA_	Dissociation constant of TIR1:IAA complex	20.86	μM^∗^	Molecular docking
*K*_D,TIR1:Ant_	Dissociation constant of TIR1:Antagonist complex	12.48	μM^∗^	Molecular docking
τ_IAN_	Cellular degradation rate of IAN	0.1	min^-1∗^	
τ_IAA_	Cellular degradation rate of IAA	0.0024	min^-1∗^	Dunlap and Robacker, 1988
τ_Antagonist_	Cellular degradation rate of antagonists	0.125	min^-1∗^	Hrncirik et al., 1998
𝜃	Production of IAA from other sources	0.00005	μmol/min^∗^	Bhalerao et al., 2002

*^∗^Value used in simulations unless explicitly stated otherwise.*

*Hyphen in reference column represents the above reference.*

For degradation rates of IAA and antagonists, we rely on *in vitro* stability assays describing the exponential decay of these compounds. The concentration of IAA is reported to remain at ∼30% of the initial concentration after 21 days which corresponds to 0.0024 min^-1^ (Dunlap et al., 1986; Dunlap and Robacker, 1988). Regarding the antagonists, a similar value is available only for the indole-3-methyl ascorbate conjugate (also known as ascorbigen). This compound shows an *in vitro* decay of 75% over the course of 10 h, which corresponds to 0.125 min^-1^ (Hrncirik et al., 1998). We apply this rate for all antagonists. Unless explicitly specified, the default parameter set was used in all simulations (**Table 1**).

### Simulations

The equations were incorporated into MATLAB and solved using the ‘ode23s’ function. The simulations were run for 3000 min with α = 0 in order to achieve steady-state. To start I3G hydrolysis, α is set to 1. For simulation of ‘mustard oil bomb’ triggering, α is set to 1 for 1 min, after which it is set to 0 and run until steady-state is achieved. To simulate the sustained cell-autonomous hydrolysis, α is set to 1 and the simulation is then run until steady-state is achieved.

### Molecular Docking

Ligands and receptors were prepared using AutoDock Tools v. 1.5.6. Docking was performed using AutoDock Vina (Trott and Olson, 2010). Grid dimensions used were 14 × 14 × 14 with a grid spacing of 1.0 Å when ligands (IAA (indol-3-acetic acid, PubChem CID: 351795), IAN (indol-3-acetonitrile, PubChem CID: 351795), I3C (indol-3-carbinol, PubChem CID: 3712), I3M-Asc (indol-3-ylmethylascorbate/ascorbigen, PubChem CID: 3081416), I3M-GSH (indol-3-ylmethylglutathione, PubChem CID: 71317122), I3M-Cys (indol-3-ylmethylcysteine, PubChem CID: 46397632), I3M-I3M-GSH (*S*-[2-(indol-3-ylmethyl)indol-3-ylmethyl]glutathione), I3M-I3M-Cys (*S*-[2-(indol-3-ylmethyl)indol-3-ylmethyl]cysteine)) were docked into the previously defined IAA binding pocket (Tan et al., 2007) or 28 × 26 × 26 to cover the entire possible binding cavity of the receptor to identify other preferred binding sites than that of the co-crystallized IAA. Docking of TIR1 in apo and bound-like states were achieved using multiple templates to reflect the different conformational states. The templates used were PDB ID: 2P1M, PDB ID: 2P1P where IAA was removed prior to docking, PDB ID: 2P1Q where IAA was removed prior to docking and PDB ID: 2P1Q where IAA and the IAA7 peptide were removed prior to docking. Evaluation of our docking strategy was performed by docking co-crystallized compounds (Tan et al., 2007; Hayashi et al., 2008) into the different templates to obtain the co-crystallized conformations.

## Results

### Dynamics Predict a Transient Drop in TIR1:IAA Concentration Upon I3G Hydrolysis

**FIGURE 2:**
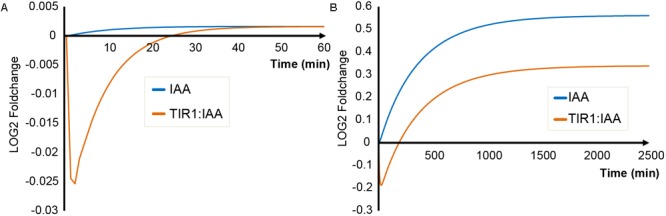
General IAA and TIR1:IAA dynamics for the two scenarios. The concentrations of model variables IAA (blue) and TIR1:IAA (orange) simulated over time, under **(A)** triggering of the ‘mustard oil bomb’ or **(B)** sustained cell-autonomous hydrolysis.

The key output of our simulations is concentration of TIR1:IAA complex as a proxy for auxin signaling events. We compare the dynamics of TIR1:IAA concentration between the two scenarios – triggering of the ‘mustard oil bomb’ and sustained cell-autonomous hydrolysis (**Figure 2**). Simulating cellular disruption (i.e., triggering of the ‘mustard oil bomb’) predicts that TIR1:IAA drops in concentration immediately after hydrolysis is initiated. This drop is then recovered over a course of 25–30 min (**Figure 2A**). Similarly, sustained I3G hydrolysis upon infection, e.g., by a biotrophic pathogen results in a rapid drop in TIR1:IAA complexes. However, recovery up to initial concentrations occurs over a course of 200 min, and the increase continues onwards for several hundreds of minutes, until a new steady-state is achieved (**Figure 2B**). The fast drop and slow recovery is due to the separation of time scales in production of antagonist and IAA; antagonist accumulates rapidly (independently of enzyme catalysis) when triggered, while the rate of IAA production is limited by the relatively low enzyme activity and abundance of NIT.

We also simulated the dynamics of IAA levels. During mustard bomb triggering, no significant alterations in IAA concentration are predicted (**Figure 2A**). Contrary to this, sustained cell-autonomous hydrolysis results in a significant increase in IAA concentration (**Figure 2B**). Another major difference between the two simulated scenarios is the relationship between IAA and TIR1:IAA dynamics. Cellular disruption attack results in a transient discoordination between IAA and TIR1:IAA that is recovered over time, whereas we observe a complete shift in the relationship between IAA and TIR1:IAA under sustained attack (**Figure 2**).

### NSP-Dependent Impact on Model Simulations of ‘Mustard Oil Bomb’ Triggering

**FIGURE 3:**
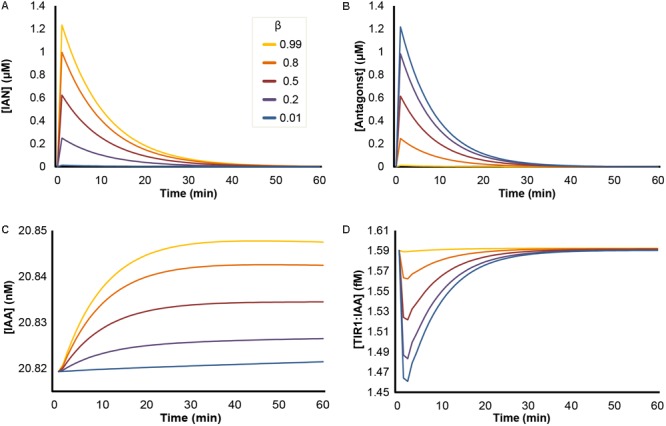
Effects of NSP-dependent hydrolysis of I3G upon ‘mustard oil bomb’ triggering. Simulation of ‘mustard oil bomb’ scenario under different β-values. Showing the concentration of dynamic variables of the model simulations: **(A)** IAN; **(B)** antagonist (an umbrella term for all ITC-derived hydrolysis products capable of binding TIR1); **(C)** IAA; and **(D)** TIR1:IAA complex.

In our initial simulations, we tested how the ratio between IAN and ITC production would influence the quantitative dynamic variables of our model upon triggering of the ‘mustard oil bomb.’ In our model, the NSP-dependent formation of IAN is annotated by the value β, ranging from 0 to 1. In Arabidopsis seedlings (Col-0), roughly 80% of GLS are converted into nitriles, corresponding to β = 0.8 (Wentzell and Kliebenstein, 2008). Simulations predict that upon attack, the concentrations of both IAN and antagonist increase rapidly, after which they follow a decay curve until they reach zero (**Figures 3A,B**). The β-value has opposing effects on the two entities, as a high β results in high conversion into IAN, and low β-values result in high levels of antagonist. Triggering of the ‘mustard oil bomb’ also results in a relative increase in IAA concentration, as the IAN will be converted into IAA by the NIT enzymes (**Figure 3C**). The rise in IAA is delayed in relation to IAN and does not reach the same amplitude. However, the rise in IAA remains after the pulse of IAN has subsided (**Figures 3A,C**). The concentration of TIR1:IAA complexes is dependent on the β-value, and shows largest drop at β = 0.01 in our simulations (**Figure 3D**). This mirrors the dynamics predicted for antagonist (**Figure 3B**), and suggests that the concentration of antagonist (and not IAA) is the major determining factor for TIR1:IAA complex formation.

### Higher Concentrations of MYR, but Not I3G, Impacts the Amplitude of Predicted TIR1:IAA Changes Upon ‘Mustard Oil Bomb’ Triggering

**FIGURE 4:**
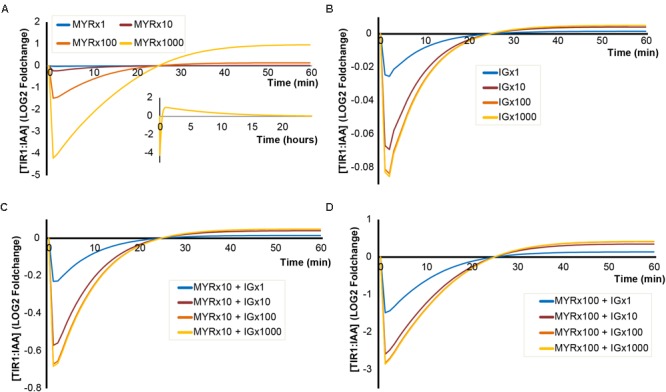
Simulated outcomes using increased concentrations of MYR and I3G. Simulated dynamics of TIR1:IAA concentrations over time at increasing concentrations of MYR and I3G. **(A)** Increased MYR concentration by 1-, 10-, 100-, and 1000-fold. **(B)** Increased I3G concentration by 1-, 10-, 100-, and 1000-fold. **(C,D)** Increased concentrations of I3G (1-, 100-, and 1000-fold) at 10- and 100-fold increased MYR concentrations, respectively.

For these simulations, we applied the concentration of MYR and I3G estimated in an intact 5-day-old seedling. However, MYR and I3G are not evenly distributed across all tissues and all cell types. At certain developmental stages, specialized protein-dense myrosin cells have been found to contain large amounts of MYR compared to surrounding tissue (Bones and Iversen, 1985; Andréasson et al., 2001). Similarly, so called S-cells have been shown to contain high amounts (>130 mM) of GLS (Koroleva et al., 2010). When breakage of adjacent myrosin and S-cells triggers the ‘mustard oil bomb,’ this results in high local concentrations of both MYR and GLS. We therefore simulated the triggering of the ‘mustard oil bomb’ at increasing concentrations of MYR and I3G, representing the higher local concentrations (**Figure 4**). A 100-fold increase in MYR predicts a more than 2-fold decrease in TIR1:IAA complex formation and a 1000-fold MYR increase predicts more than 16-fold decrease in complex formation (**Figure 4A**). A noteworthy feature of high MYR concentration is the apparent ‘overshot’ in the recovery (**Figure 4A**, insert). This results in a TIR1:IAA peak roughly 60 min after hydrolysis is initiated, which eventually falls back into the original steady-state over the course of 24 h.

**FIGURE 5:**
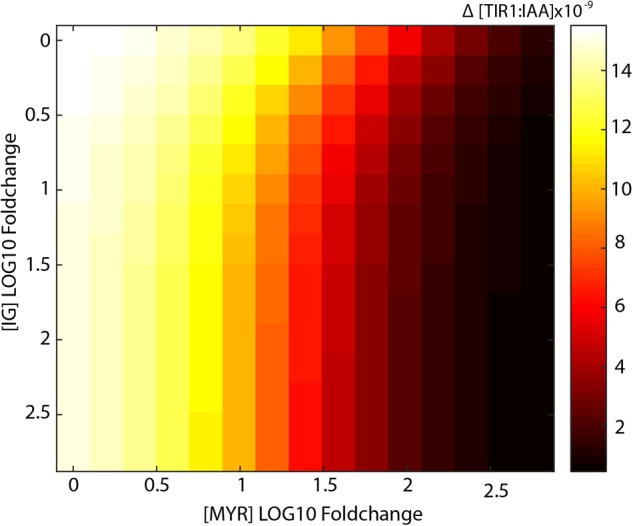
Synergistic effects of increased I3G and MYR on the drop in TIR1:IAA concentration. Heatmap showing the amplitude of the transient drop in [TIR1:IAA] (μM) achieved by increasing concentrations of both MYR and I3G.

Increased levels of I3G, however, do not result in large decreases in TIR1:IAA complex formation. Even at more than hundred-fold increased I3G concentration, we do not observe a notable inhibition of TIR1:IAA complex formation (**Figure 4B**). As it is possible that the two components, MYR and I3G, have a synergistic relationship, we tested the effect of increasing the concentration in both variables in our model. At 10-fold higher MYR, we find that increased I3G is able to further reduce TIR1:IAA complex formation (**Figure 4C**). This suggested synergistic effect is even clearer at a 100-fold increased MYR concentration, where the combination with 10-fold increased I3G leads to an increased inhibition from 3-fold to 6-fold (**Figure 4D**). The simulated effect of increased I3G stagnates at higher I3G concentrations, but increases with higher concentrations of MYR (**Figure 5**). It would thus seem that MYR is a major factor in determining the dynamics of I3G hydrolysis and its impact on auxin signaling.

### Simulations Predict That Nitrilases Specifically Affect the TIR1:IAA Recovery Rate and Pulse Formation

**FIGURE 6:**
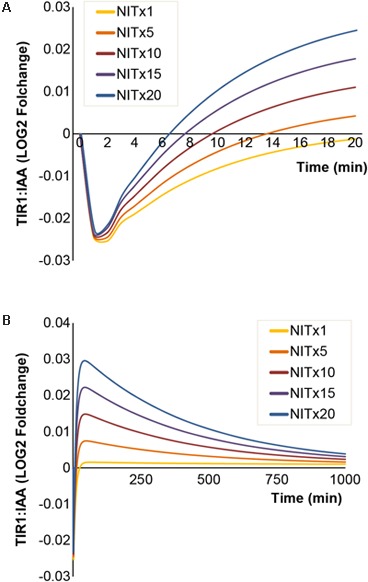
Increasing NIT concentrations result in faster [TIR1:IAA] recovery rates and amplitude of overshot. The simulated dynamics of [TIR1:IAA] under increasing concentrations of NIT. **(A)** Simulated drop in [TIR1:IAA] remains unchanged at increasing concentrations of NIT, but recovery rates of [TIR1:IAA] increase. **(B)** Increased recovery rates result in larger positive pulse formation at higher NIT concentrations.

Besides the MYR enzymes covered above, the NIT enzymes are also included in our model. These enzymes are capable of converting IAN to IAA, thus potentially allowing a positive enforcement of auxin signaling upon I3G hydrolysis. We simulated the effects of increasing concentrations of NIT and find that the drop in TIR1:IAA concentration is not affected by the NIT concentrations tested (**Figure 6A**). However, the recovery time for re-establishing baseline TIR:IAA complex levels decreases at higher NIT concentrations (**Figure 6A**). The simulations predict that higher concentrations of NIT affect the ‘overshot’ described above (**Figure 6B**). However, contrary to the effects seen for MYR, changing NIT concentration had a predicted impact only on the overshot and not the amplitude of the drop (**Figure 6**).

### Simulated Dynamics of the Sustained Hydrolysis Scenario

**FIGURE 7:**
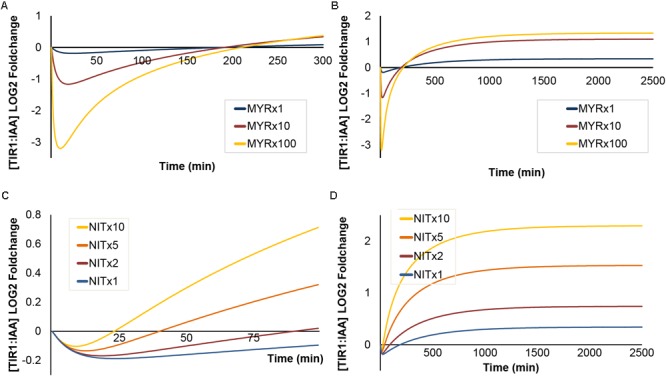
The effects of increased [MYR] and [NIT] under sustained I3G hydrolysis. Simulated outcomes of increasing concentration of MYR (1-, 10-, and 100-fold) under sustained I3G hydrolysis shown on a short **(A)** and long timescale **(B)**. Increased NIT (1-, 10-, and 100-fold) under sustained I3G hydrolysis shown on a short **(C)** and long timescale **(D)**.

So far we have examined the dynamics of the ‘mustard oil bomb’ scenario on TIR:IAA concentration. In the following, we will test the dynamics of sustained, cell-autonomous hydrolysis of I3G that occurs upon biotrophic pathogen infection. We set out to test whether sustained I3G hydrolysis is influenced by variations in the concentration of the MYR and NIT enzymes. First, increasing MYR predicts a similar trend as for brief attack, with high MYR concentrations resulting in a severe drop in TIR1:IAA concentration, followed by a recovery and an overshot (**Figures 7A,B**). However, the ‘overshot’ does not lead to a peak but rather a new steady-state for TIR1:IAA under sustained hydrolysis. The simulated effect of increased I3G concentration mirrors our observations from ’mustard oil bomb’ triggering, and does not change the overall dynamics (Supplementary Figure S1). Similar to what is observed under ‘mustard oil bomb’ triggering, increasing NIT concentration does not predict changes in the amplitude of the TIR1:IAA drop notably, but increases recovery rate significantly (**Figure 7C**). The increased recovery rate results in a larger overshot and – as seen for MYR – defines a new steady-state by forming a plateau rather than a peak (**Figure 7D**). Sustained I3G hydrolysis and triggering of the mustard oil bomb thus appear to have distinct dynamics in our model.

### Enzyme Isoforms Define the Simulated Impact of I3G Hydrolysis

**FIGURE 8:**
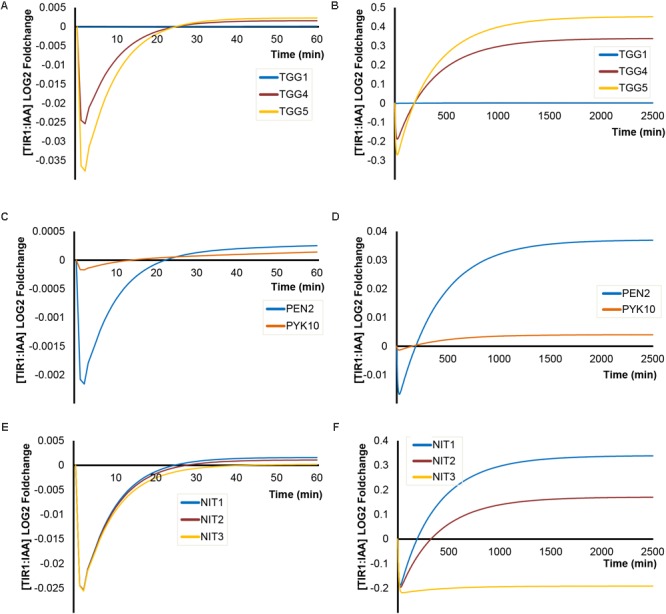
Impact of enzyme isoforms on [TIR1:IAA] dynamics. Simulations of [TIR1:IAA] over time for **(A)** different classical myrosinase isoforms under ‘mustard oil bomb’ triggering and **(B)** under sustained I3G hydrolysis; **(C)** atypical myrosinases under ‘mustard oil bomb’ triggering and **(D)** sustained hydrolysis; **(E)** different NIT isoforms under ‘mustard oil bomb’ triggering **(F)** and sustained hydrolysis.

For both of the enzymatic steps catalyzed by MYR and NIT, several known enzyme isoforms exist with different kinetic parameters (**Table 1**). To get an overview of the potential impact of the enzyme isoforms on TIR1:IAA complex formation, we ran simulations using their individual kinetic parameters and estimated abundance. We find large differences in their influence on the dynamics of TIR1:IAA. For MYR enzymes, we find that the classical MYRs TGG4 and TGG5 differ slightly – TGG5 being more effective – and that both enzymes are capable of eliciting the predicted inhibitory effects followed by an overshot and subsequent higher steady-state under ‘mustard oil bomb’ triggering or sustained hydrolysis (**Figures 8A,B**). TGG1, on the other hand, seems to have negligible effect compared to TGG4 and TGG5. This corresponds with the markedly lower kinetic rates and estimated abundance of TGG1 compared with TGG4 and TGG5 (**Table 1**). In addition to the classical MYRs, we also simulated the atypical MYR PEN2 which has been shown to influence pathogen susceptibility via, e.g., inducing callose deposition (Bednarek et al., 2009; Clay et al., 2009) and PYK10 which is similarly described to be involved in plant defense (Nitz et al., 2001; Nakano et al., 2017). Both PEN2 and PYK10 are, relative to the classical MYRs, low kinetic-rate enzymes that display little capacity to effect TIR1:IAA concentration (**Figures 8C,D**).

Comparing the three enzymes NIT1, NIT2, and NIT3 [NIT4 is excluded as it does not convert IAN to IAA (Dohmoto et al., 2000; Piotrowski et al., 2001)], our simulations predict very little differences in the kinetic properties under bomb triggering (**Figure 8E**). However, NIT3 seems to be unable to recover the drop in TIR1:IAA under sustained hydrolysis (**Figure 8F**), whereas the two enzymes NIT1 and NIT2 efficiently recover the drop and establish a new higher steady-state (**Figure 8E**). It appears that the properties of the specific enzyme isoforms (i.e., abundance, *V*_max_ and *K*_M_) give rise to simulated dynamics of varying intensity.

### Indol-3-Ylmethyl Conjugates as Auxin Antagonists

The ITC product of I3G breakdown is highly reactive and readily reacts with abundant nucleophilic molecules of the cell (e.g., cysteine, ascorbate, and glutathione) forming products other than I3C (Agerbirk et al., 1998, 2009; Staub et al., 2002). These reactions result in a series of I3M conjugates, some of which are known to self-react and form dimeric indole-indole-conjugates (such as I3M-I3M-cysteine and I3M-I3M-glutathione) (Agerbirk et al., 1998, 2009; Staub et al., 2002).

**FIGURE 9:**
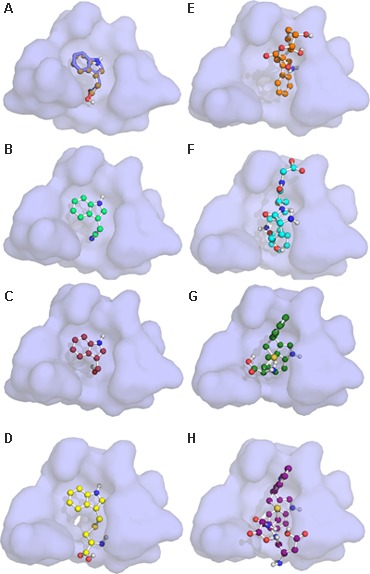
Molecular docking of I3G hydrolysis products onto the TIR1 receptor. The auxin binding pocket of TIR1 with the docked molecules: **(A)** IAA; **(B)** IAN; **(C)** I3C; **(D)** I3M-cysteine; **(E)** I3M-ascorbate; **(F)** I3M-glutatione; **(G)** I3M-I3M-cysteine; and **(H)** I3M–I3M-glutatione in ball-and-stick representation. Red spheres represent oxygen atoms, blue spheres represent nitrogen atoms and dark yellow spheres represent sulfur atoms. The blue stick-representation molecule of **(A)** shows the IAA position of the crystal structure. The lowest energy binding state is seen in **(A–D)**, whereas **(E,G,H)** display the second lowest energy state, and **(F)** displays the third lowest energy binding state of the observed conformations shown in Supplementary Figure S1.

Auxin signaling involves active degradation of regulatory proteins after auxin binds to the receptor TIR1. The IAA7 protein is an example of such a regulatory protein, which is degraded upon interaction with the TIR1:IAA complex (Gray et al., 2001). Efficient inhibition of auxin signaling would require that a competitive auxin antagonist not only binds to TIR1 instead of IAA, but also blocks the further interaction between TIR1 and the regulatory protein (i.e., IAA7). Taking advantage of the published crystal structure of the TIR1 receptor (Tan et al., 2007; Hayashi et al., 2008; Strader and Zhao, 2016), we used molecular docking simulations to examine whether IAA, IAN, I3C and five different I3M conjugates can bind to the TIR1 receptor and inhibit its interaction with IAA7. Molecular docking simulations of IAA, IAN, and I3C were recently reported by another research group (Katz et al., 2015b), but none of the compounds were docked to the IAA binding pocket in the published crystal structure (Supplementary Figure S2). Our docking results predict that all the molecules are capable of binding to the auxin binding pocket of the published TIR1 structure (**Figure 9**). Based on these docking simulations we find that I3C is a relatively weak binder of TIR1 with a dissociation constant (*K*_D_) more than twice that of IAA (**Table 2**, left column). However, our calculations predict that many of the conjugates bind strongly to the free TIR1 receptor. This suggests that they could effectively block IAA binding and therefore serve as competitive auxin antagonists (**Table 2**, left column).

**Table 2:** Estimated dissociation constants (μM^-1^).

Molecule	TIR1	TIR1:IAA7
IAA	20.86	1.35
IAN	29.37	1.60
I3C	49.06	6.30
I3M-cysteine	24.75	6.30
I3M-ascorbate	12.48	115.41
I3M-glutathione	6.30	69.08
I3M–I3M-cysteine	14.81	81.97
I3M–I3M-glutathione	1.14	1.11^∗^10^9^

**FIGURE 10:**
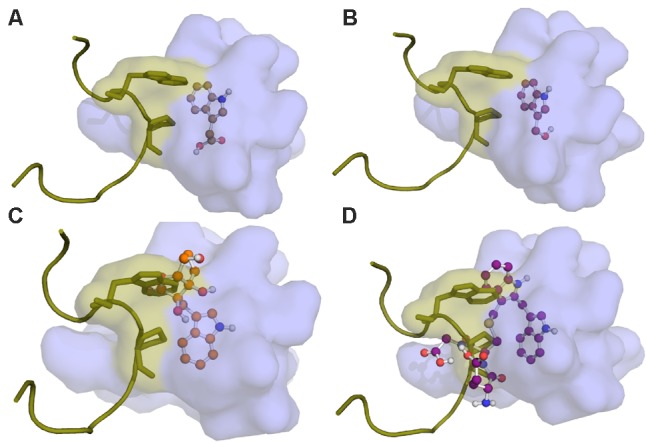
Molecular docking of I3G hydrolysis products onto the TIR1 receptor and subsequent IAA7 superimposition. The auxin binding pocket of TIR1 is shown in light blue. The IAA7 peptide is represented in yellow. Predicted binding conformations of **(A)** IAA; **(B)** I3C; **(C)** I3M-ascorbate; and **(D)** I3M-I3M-glutathione in the TIR1 receptor is presented in ball-and-stick.

**FIGURE 11:**
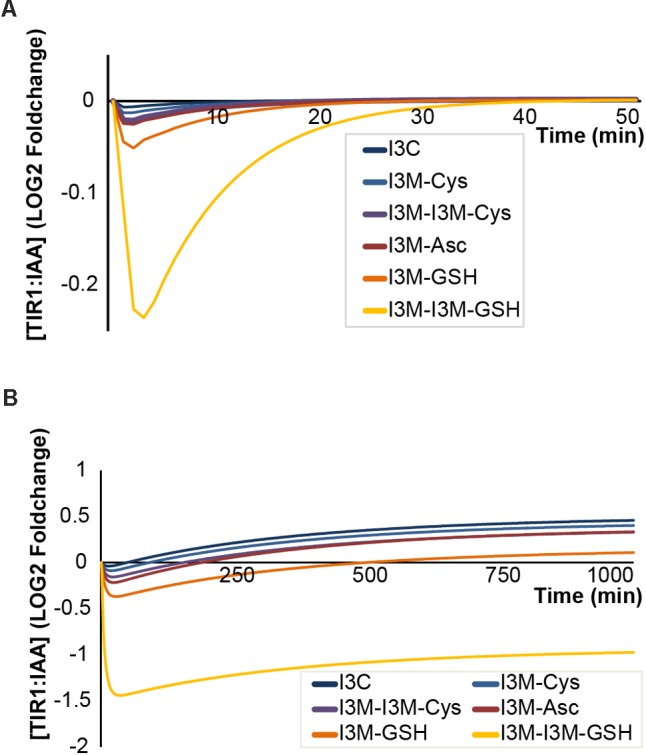
Antagonistic properties of I3C and I3M-conjugates on TIR1:IAA complex formation. Effect of different antagonists under **(A)** triggering of the ‘mustard oil bomb’ or **(B)** sustained cell-autonomous hydrolysis. Colors from blue to yellow indicate higher affinity of antagonist. Cys, cysteine; Asc, ascorbate; and GSH, glutathione.

In the molecular docking simulations, we observe that IAA7 encounters spatial hindrance by several of the I3M conjugates, and that *K*_D_ of the conjugates are high relative to IAA in the TIR1:IAA:IAA7 complex, which suggest that the conjugates cannot be accommodated in the binding cavity between TIR1 and IAA7 (**Table 2**, right column and **Figure 10**). Contrary to this, the *K*_D_ of IAA, which drops from 20.86 to 1.35 μM^-1^ as IAA7 binds the TIR1:IAA complex, suggests a tighter fit of IAA in the binding pocket between TIR1 and IAA7 compared to TIR1 alone. This lower energy is supported by the emergence of T-shaped π-stacking between the tryptophan of IAA7 and the indole ring of IAA. For the conjugates – and especially I3M-I3M-glutatione – we observe an increase in *K*_D_ upon IAA7 binding, which implies that the conjugates block the interaction between TIR1 and the regulatory protein, IAA7. We then simulated the effect of the various conjugates (with varying binding affinities, **Table 2** left column) on auxin signaling upon triggering of the ‘mustard oil bomb’ as well as cell-autonomous hydrolysis. There is significantly larger effect of high-affinity conjugates on TIR1:IAA concentration, with the I3M–I3M-glutathione conjugate showing the biggest effect (**Figures 11A,B**).

## Discussion

In this study, we constructed a mathematical model to investigate dynamics of I3G hydrolysis and its impact on auxin signaling. We used our model to simulate the effects of I3G hydrolysis on auxin signaling during two scenarios – a cellular disruptive attack leading to triggering of the ‘mustard oil bomb’ and under sustained cell-autonomous hydrolysis of I3G in intact cells. The output of our simulations under the two scenarios is – besides concentrations of metabolites, i.e., I3G, IAA, IAN and antagonists – changes in TIR1:IAA complex concentration and thereby auxin signaling.

### Characteristic Features of the Proposed Model

In the simulations, the main descriptors of the TIR1:IAA response include: (i) the transient drop in TIR1:IAA concentration, (ii) the time it takes for recovery from this drop and (iii) the pronounced overshot which can result in either a transient pulse or a redefined steady-state. These features are common for both sustained cell-autonomous hydrolysis and ‘mustard oil bomb’ triggering, but are controlled by different parameters. Our model predicts that the drop in TIR1:IAA concentration depends on the amounts of produced antagonist – which depends on MYR kinetics and abundance, I3G concentration and NSP activity – and on how efficiently the antagonist binds to TIR1, which depends on *K*_D_ of the respective antagonist. The amplitude of the TIR1:IAA drop is severely affected by varying concentrations of MYR and binding affinities of the antagonists to TIR1.

The time to recovery from drop to steady state is defined by replacement of antagonist by IAA in the TIR1 binding pocket. In our simulations, this was affected by MYR and I3G concentration as well as the binding affinity of the antagonist. Noticeably, the recovery phase could be manipulated by changing the concentration of NIT, and this effect is achieved without affecting the amplitude of the transient drop in TIR1:IAA. The dynamics of recovery phase differ depending on our two scenarios. A brief attack detonating the mustard bomb results in a transient drop in TIR1:IAA complexes with a quick recovery phase of 30 min. Under sustained hydrolysis, the recovery phase occurs over a period of several hundred minutes, unless NIT concentration is increased. Under increased NIT levels, the recovery times are shortened significantly in both attack scenarios. Our simulations suggest that the NIT enzymatic activity is important for defining the homeostasis of auxin signaling after I3G hydrolysis.

The steady state level of TIR1:IAA complexes after I3G hydrolysis differs between the two attack scenarios. The ‘mustard oil bomb’ results in a fast drop in the TIR1:IAA complex level followed by an overshot resulting in a peak that collapses into the original steady-state. Sustained cell-autonomous hydrolysis, on the other hand, does not go back to the original steady-state level but rather establishes a new steady-state dependent on enzyme concentration and binding affinity of the antagonist. The overshot reflects an increased amount of free IAA available to bind to free TIR1 receptors. This is – similar to the recovery phase – affected by MYR and I3G, but primarily by NIT that boosts the production of IAA from I3G hydrolysis. Our model thereby displays distinct dynamics in TIR1:IAA complex levels depending on ‘mustard oil bomb’ triggering or sustained cell-autonomous hydrolysis.

### Attribution of Enzyme Abundance and Kinetics on the Model Outcome

Our simulations of I3G hydrolysis and its impact on auxin signaling show that MYR abundance and activity are critical in determining the initial drop in TIR1:IAA concentration. At averaged MYR concentrations across the seedling, we see very little effect of I3G hydrolysis. However, it is well-established that in species related to Arabidopsis, MYR is concentrated in specific idioblast cells known as myrosin cells that constitute 1–4% of total cells (Bones and Iversen, 1985; Andréasson et al., 2001). In our model, MYR is assumed to be equally distributed at an average concentration throughout the plant. Around the myrosin cells, however, the local concentration of MYR can reach as much as 100-times the average concentration. Similarly, GLS are not equally distributed throughout the plant, but rather are concentrated at strategically important sites (Halkier and Gershenzon, 2006; Wittstock and Burow, 2010; Madsen et al., 2014). Particularly one cell type, the S-cells, has received attention due to its high GLS content. These cells have been reported to contain as much as 8 mM I3G (Koroleva et al., 2010). S-cells and myrosin cells are in close proximity to each other (Andréasson et al., 2001), and triggering of the ‘mustard oil bomb’ will thus result in the release of high concentrations of MYR and GLS. Our model shows that the concentration of I3G has only a minor influence, and that it increases the amplitude of the drop in TIR1:IAA concentration, but only up to 50-fold increased concentration, after which the effect stagnates. This feature would allow high accumulations of I3G with no significant effect on the auxin signaling dynamics. Our simulations suggest that increased MYR rather than I3G concentrations can have substantial potential to affect TIR1:IAA concentration upon triggering of the ‘mustard oil bomb.’

Regarding the NITs, NIT1 and NIT2 give rise to strong dynamics, whereas NIT3 seem unable to elicit a strong effect. All NIT enzymes are generally lowly expressed in unchallenged tissue (Grsic-Rausch et al., 2000; Kutz et al., 2002; Lehmann et al., 2017). However, all three isoforms display inducible expression (Grsic-Rausch et al., 2000; Kutz et al., 2002; Cao et al., 2016; Lehmann et al., 2017). As much as 30-fold increase in all three NIT transcripts was observed 8 h after treatment with MeJa (Cao et al., 2016). We speculate based on our model that induced NIT gene expression would provide a longer period with low TIR1:IAA complex formation followed by a rapid recovery and increased steady-state level.

Nitrile-specifier protein activity levels in Arabidopsis vary among naturally occurring accessions, organs and developmental stages (Wentzell and Kliebenstein, 2008; Burow and Wittstock, 2009; Wittstock et al., 2016). While seedlings of Arabidopsis Col-0 predominantly produce nitriles, disruption of rosette tissue of the same genotype results in the formation of primarily ITCs. Transcripts encoding specifier proteins can be upregulated upon jasmonates treatment and herbivory (Burow and Wittstock, 2009. In Arabidopsis Col-0, induction of *NSP1* leads to nitrile formation in rosette leaves infested larvae of *Pieris rapae* (Burow et al., 2009). Switching between different types of GLS activation products reflects the ability of the plant to adjust its chemical defense to changes in the biotic environment, and based on our model, this may also include differential regulatory input to the auxin signaling network.

Besides the impact of enzyme abundance, the various isoforms of the enzymes elicit significant difference in the inhibitory effects on TIR1:IAA complex dynamics. For the MYR enzymes, we see strong dynamics with TGG4 and TGG5, but comparatively little effect of TGG1. The *V*_max_ of TGG1 is the lowest of the three TGGs, and it is found at low abundance in root tissue – the source tissue we have used to estimate absolute protein abundance. Together, these factors explain the behavior seen with the TGG1 enzyme. It is possible that the higher expression in aerial tissue will result in larger effects for this enzyme. This opens up for a tissue-specific parameter regime, an aspect not covered in the presented work. The atypical MYRs PEN2 and PYK10 are unable to produce the strong dynamics seen for TGG4 and TGG5. However, as both of these enzymes are thought to be involved in sustained cell-autonomous hydrolysis, their relative low kinetic rates could reflect a desired parameter regime for exactly this situation. PEN2 and PYK10 would not give rise to a large drop (and the subsequent overshot) in TIR1:IAA complex formation, but would allow for more fine-tuned influence on auxin signaling. In summary, our simulations address the role of various enzyme isoforms in defining the physiological output and show that each enzyme represents a specific parameter regime, which on its own or in combination with its isoforms would allow for fine-tuning the required response at specific sites.

### Auxin Antagonism by I3G Hydrolysis Products

When the potential of I3G hydrolysis products as antagonists in auxin signaling was investigated by molecular docking simulations using the crystal structure of TIR1, we were able to dock IAA, IAN, I3C and multiple I3M-conjugates to the auxin binding site of TIR1 (Tan et al., 2007). The binding affinities based on these dockings showed a capacity for the I3M-conjugates to bind tighter to the free TIR1 receptor as compared to IAA, IAN, and I3C, making them potential strong antagonists of IAA. Notably, the previous reported IAA antagonist, I3C, seems an inefficient antagonist based on its low binding affinity relative to IAA.

As auxin signaling is mediated through the IAA-facilitated interaction between TIR1 and the IAA7 regulatory protein, we superimposed IAA7 onto the TIR1:IAA complex. Here we see that IAA has a higher binding affinity to the complex, suggesting that IAA fits tightly into its binding pocket and facilitates TIR1:IAA:IAA7 complex formation. On the contrary, the I3M conjugates show a severe decline in theoretical binding affinity to the TIR1 receptor in the presence of IAA7. Together with the molecular docking structures – which display clear overlap between I3M conjugates and the sidechains of IAA7 (**Figures 10C,D**) – our data suggest that these small molecules do not fit into the binding cavity between TIR1 and IAA7 and thereby disturb the protein complex formation due to sterical hindrance. The correlation between bulky molecular structure and auxin antagonism is supported by studies using synthetic auxin-antagonists, which showed that bulky auxin analogs are efficient auxin antagonists (Hayashi et al., 2008, 2012). However, a large molecular structure could result in slow diffusion and hamper cell-permeability – aspects that are not considered in our current model.

Our model simulations indicate that all I3G hydrolysis products are able to bind to the TIR1 receptor and interfere with TIR1:IAA complex formation, but to different extents. The strength of the inhibition of TIR1:IAA complex formation (monitored as drop in TIR1:IAA level upon attack) correlates with the dissociation constants; higher affinity results in stronger inhibition. It is important to point out that the I3M-conjugates used here represent just a small part of possible chemical complexity that can arise upon hydrolysis of I3G (Kim et al., 2008; Agerbirk et al., 2009; Bednarek et al., 2009). It is possible that a highly complex mixture of these compounds emerges upon attack, and that this mixture can be modulated to achieve a specific dynamic response which is not fulfilled by any single compound. However, the I3M-ascorbate conjugate (also known as ascorbigen) is the predominant end-product of indole ITC from crushed tissue (McDanell et al., 1987; Preobrazhenskaya et al., 1993). Ascorbigen arises when the unstable indole ITC reacts with ascorbic acid, which is highly abundant within plant tissue. In addition, ascorbic acid has been shown to modulate the catalytic efficiency of some classical MYRs (Andersson et al., 2009). Including ascorbate in future iterations of our model could help shed light on its role in I3G hydrolysis.

The high-affinity glutathione conjugates are also of special interest, as they appear to be able to very effectively uncouple auxin signaling from the IAA concentration. Conjugation of glutathione to metabolites is a classic detoxification strategy, but it appears that glutathione conjugation is tightly linked with defense chemistry in cruciferous plants, as it is critical for biosynthesis of GLS (Halkier and Gershenzon, 2006; Schlaeppi et al., 2010; Geu-Flores et al., 2011) as well as the conjugation of the reactive ITCs that result from GLS hydrolysis (Bednarek et al., 2009; Klein and Sattely, 2017). Like ascorbate, glutathione is highly abundant in plant tissue, and I3G hydrolysis likely results in large amounts of the high-affinity I3M-glutathione conjugates. This would predict a large transient drop in TIR1:IAA complex level and thereby auxin signaling upon I3G hydrolysis.

### Considerations on the Initial Assumptions

As described in Section “Mathematical Modeling” we had to make a series of assumptions in order to build the model. These assumptions were made to either compensate for a current lack of knowledge or an attempt to simplify highly complex subsystems, as to enable modeling in the first place. Assumptions: 2, 5, and 7 all seek to mitigate the problem of missing experimental data on biochemical and biophysical properties of the molecules modeled in this manuscript. The assumptions 2 and 5, which cover the *on*-rate of TIR1:IAA and degradation rate of IAN, are not expected to influence the dynamics greatly based on our simulations. It can, however, be expected that these rates might affect the timing of the dynamics slightly. We further assumed that MYR activity toward I3G is the same as for sinigrin (Assumption 7). Supporting the assumption that the side chain chemistry does not have a large impact on MYR kinetics, no notable differences in MYR activities were found in crude extracts of Brassica vegetables in assays comparing sinigrin and the aromatic benzylglucosinolate (Piekarska et al., 2013). Yet, one of the key findings of this manuscript is that enzyme properties and antagonist binding are major factors in determining the simulated dynamics, and it is reasonable to believe that even slight changes in MYR activity will lead to relatively large changes in the simulated dynamics, as is seen for typical and atypical MYR. However, we consider the enzyme properties as major factors that allow for distinct parameter regimes. Rather than depicting a strictly quantitative prediction, the current simulations survey the dynamic patterns and trends that are inherent to the model. For strictly quantitative predictions, kinetics for MYR hydrolysis of I3G will be needed.

Assumptions 1, 3, 4, 6, and 8 are efforts made to simplify the biological and chemical complexity of I3G hydrolysis and auxin signaling, which makes it difficult to speculate on the specific effects of these assumptions on the presented model. This would require actual modeling of the individual – something that we expect to become feasible in the future. Finally, it is important to note that the model of I3G hydrolysis and auxin signaling presented here is not considered final and absolute, but rather serves as a concrete hypothesis, which must be challenged experimentally, and modified accordingly. Ultimately, the model should be extended to cover more complex phenomena as models of these will emerge in the future.

### Physiological Insights and Hypotheses

When inflicted with a brief attack – as tissue disruption upon herbivore feeding or a necrotrophic pathogen infection – the ‘mustard oil bomb’ is triggered, leading to the release of multiple GLS hydrolysis products. We simulated here specifically the influence of I3G hydrolysis products on auxin signaling and predict that upon triggering the ‘mustard oil bomb,’ a burst of I3M conjugates is released from the attack site which can cause a dramatic, but very transient drop in the abundance of TIR1:IAA complexes (and thereby auxin signaling events). This drop is followed by a steep recovery phase, and eventual an overshot in TIR1:IAA complex levels, resulting in a peak roughly 60 min after I3G hydrolysis is initiated.

In addition to the changes in TIR1:IAA complex levels, our model simulations show increasing concentrations of IAA upon I3G hydrolysis. Interestingly, recent findings in tobacco showed a similar, rapid increase in auxin after herbivore attack (Machado et al., 2016). However, as tobacco does not contain I3G it cannot be a shared mechanism between the two species. A similar (but slower) increase in auxin upon herbivore attack is reported in maize (Maag et al., 2016). Increased levels of auxin in response to herbivory could therefore be general plant behavior across species. It is possible that I3G-producing plants have been under positive selection pressure to contribute to this auxin peak upon attack, whereas non-GLS-producing plants, like tobacco, rely entirely on other IAA sources such as the YUCCA pathway (Machado et al., 2016).

During sustained cell-autonomous I3G hydrolysis, e.g., under infection by certain biotrophic pathogens (Bednarek et al., 2009; Clay et al., 2009), our model simulations show a similar drop in TIR1:IAA complex levels upon attack. However, with strong antagonists, e.g., I3M-glutathione (and in absence of adequate NIT enzyme activity) the drop is not recovered, but instead maintained at a new lower level. We hypothesize that this could be a strategy allowing the plant to cope with auxin derived from the pathogen. In this scenario the I3M conjugates help maintain the concentration of TIR1:IAA (and thus auxin signaling) at a relatively low level – which does not favor pathogen infection – independent of the actual IAA concentration. The low enzyme kinetic properties of PEN2 and PYK10 could enforce this uncoupling between IAA concentration and TIR1:IAA complex formation, as they enable the emergence of strong antagonists but limited amount of IAN.

There are several reports of pathogens manipulating plants by modulating their auxin levels, e.g., weakening of the cell-wall and inhibition of SA-associated defenses (Yamada, 1993; Fu and Wang, 2011; Huot et al., 2014; Chanclud and Morel, 2016). The incoherent feedforward loop presented here suggest that Arabidopsis (and possibly other *Brassicacea* species) may have evolved mechanisms do counteract such auxin-modulating pathogens. Whether auxin-modulating pathogens occur on *Brassicacea* species with higher frequency than on non-GLS species, remains to be investigated. However, *Plasmodiophora brassicae* which causes clubroot disease in *Brassicacea* species and results in large crop losses (Ludwig-Müller and Schuller, 2008; Diederichsen et al., 2009), is a pathogen that forms large galls on the roots by manipulating the auxin levels of its host (Ludwig-Müller et al., 2009). It has been suggested that the auxin mobilized by clubroot originate from IG, however, experimental evidence suggests that there is no simple relationship between IG hydrolysis and clubroot disease (Ludwig-Müller et al., 1999). The model presented here would suggest that *P. brassicae* as a specialist pathogen evolved means to specifically disrupt the feedforward loop described in this manuscript, either by increasing the IAN-to-I3C ratio upon hydrolysis or by directly converting antagonists into IAA, thereby effectively removing the uncoupling between IAA levels and auxin signaling events.

Through simulations using our mathematical model for I3G hydrolysis and its impact on auxin signaling, we have theoretically examined the possible outcomes of the system. Further experimental exploration of the direct relation between I3G hydrolysis and auxin signaling is required to validate it. The model should be viewed as a research tool that allows for early exploration and hypothesis generation.

## Author Contributions

DV and MB outlined the regulatory network. DV and NM defined and formulated the equations. NW performed the molecular docking simulations. DV simulated the model and analyzed the data. DV wrote the manuscript supported by MB and BH.

## Conflict of Interest Statement

The authors declare that the research was conducted in the absence of any commercial or financial relationships that could be construed as a potential conflict of interest.

## Abbreviations

GLS: glucosinolate
I3C: indole-3-carbinol
I3G: indol-3-ylmethyl glucosinolate
I3M: indol-3-ylmethyl
IAA: indole-3-aceticacid
IAN: indole-3-acetonitrile
ITC: indole-3-acetoisothiocyanate
MYR: myrosinase
NIT: nitrilase
NSP: nitrile-specifier protein

## References

[B1] Agerbirk N. De Vos M. Kim J. H. Jander G. (2009). Indole glucosinolate breakdown and its biological effects. *Phytochem. Rev.* 8 101–120. 10.1007/s11101-008-9098-0

[B2] Agerbirk N. Olsen C. E. Sørensen H. (1998). Initial and final products, nitriles, and ascorbigens produced in myrosinase-catalyzed hydrolysis of indole glucosinolates. *J. Agric. Food Chem.* 46 1563–1571. 10.1021/jf9708498

[B3] Alon U. (2007). Network motifs: theory and experimental approaches. *Nat. Rev. Genet.* 8 450–461. 10.1038/nrg2102 17510665

[B4] Andersson D. Chakrabarty R. Bejai S. Zhang J. Rask L. Meijer J. (2009). Myrosinases from root and leaves of *Arabidopsis thaliana* have different catalytic properties. *Phytochemistry* 70 1345–1354. 10.1016/j.phytochem.2009.07.036 19703694

[B5] Andréasson E. Bolt Jørgensen L. Höglund A. S. Rask L. Meijer J. (2001). Different myrosinase and idioblast distribution in Arabidopsis and *Brassica napus*. *Plant Physiol.* 127 1750–1763. 10.1104/pp.010334 11743118 PMC133578

[B6] Baerenfaller K. Hirsch-Hoffmann M. Svozil J. Hull R. Russenberger D. Bischof S. (2011). pep2pro: a new tool for comprehensive proteome data analysis to reveal information about organ-specific proteomes in *Arabidopsis thaliana*. *Integr. Biol.* 3 225–237. 10.1039/c0ib00078g 21264403

[B7] Bednarek P. Pislewska-Bednarek M. Svatos A. Schneider B. Doubsky J. Mansurova M. (2009). A glucosinolate metabolism pathway in living plant cells mediates broad-spectrum antifungal defense. *Science* 323 101–106. 10.1126/science.1163732 19095900

[B8] Beekwilder J. van Leeuwen W. van Dam N. M. Bertossi M. Grandi V. Mizzi L. (2008). The impact of the absence of aliphatic glucosinolates on insect herbivory in Arabidopsis. *PLoS One* 3:e2068. 10.1371/journal.pone.0002068 18446225 PMC2323576

[B9] Bhalerao R. P. Eklöf J. Ljung K. Marchant A. Bennett M. Sandberg G. (2002). Shoot-derived auxin is essential for early lateral root emergence in *Arabidopsis* seedlings. *Plant J.* 29 325–332. 10.1046/j.0960-7412.2001.01217.x 11844109

[B10] Bones A. Iversen T.-H. (1985). Myrosin cells and myrosinase. *Isr. J. Bot.* 34 351–376.

[B11] Burow M. Losansky A. Müller R. Plock A. Kliebenstein D. J. Wittstock U. (2009). The genetic basis of constitutive and herbivore-induced ESP-independent nitrile formation in Arabidopsis. *Plant Physiol.* 149 561–574. 10.1104/pp.108.130732 18987211 PMC2613743

[B12] Burow M. Markert J. Gershenzon J. Wittstock U. (2006). Comparative biochemical characterization of nitrile-forming proteins from plants and insects that alter myrosinase-catalysed hydrolysis of glucosinolates. *FEBS J.* 273 2432–2446. 10.1111/j.1742-4658.2006.05252.x 16704417

[B13] Burow M. Wittstock U. (2009). Regulation and function of specifier proteins in plants. *Phytochem. Rev.* 8 87–99. 10.1007/s11101-008-9113-5

[B14] Cao J. Li M. Chen J. Liu P. Li Z. (2016). Effects of MeJA on *Arabidopsis* metabolome under endogenous JA deficiency. *Sci. Rep.* 6:37674. 10.1038/srep37674 27883040 PMC5121592

[B15] Chanclud E. Morel J. B. (2016). Plant hormones: a fungal point of view. *Mol. Plant Pathol.* 17 1289–1297. 10.1111/mpp.12393 26950404 PMC6638337

[B16] Clay N. K. Adio A. M. Denoux C. Jander G. Ausubel F. M. (2009). Glucosinolate metabolites required for an Arabidopsis innate immune response. *Science* 323 95–101. 10.1126/science.1164627 19095898 PMC2630859

[B17] Csikász-Nagy A. Kapuy O. Tóth A. Pál C. Jensen L. J. Uhlmann F. (2009). Cell cycle regulation by feed-forward loops coupling transcription and phosphorylation. *Mol. Syst. Biol.* 5 815–826. 10.1038/msb.2008.73 19156128 PMC2644172

[B18] de Vos M. Kriksunov K. L. Jander G. (2008). Indole-3-acetonitrile production from indole glucosinolates deters oviposition by *Pieris rapae*. *Plant Physiol.* 146 916–926. 10.1104/pp.107.112185 18192443 PMC2259081

[B19] Diederichsen E. Frauen M. Linders E. G. A. Hatakeyama K. Hirai M. (2009). Status and perspectives of clubroot resistance breeding in crucifer crops. *J. Plant Growth Regul.* 28 265–281. 10.1007/s00344-009-9100-0

[B20] Dohmoto M. Tsunoda H. Isaji G. Chiba R. Yamaguchi K. (2000). Genes Encoding Nitrilase-like Proteins from Tobacco. *DNA Res.* 7 283–289. 10.1093/dnares/7.5.283 11089910

[B21] Dunlap J. R. Kresovich S. McGee R. E. (1986). The effect of salt concentration on auxin stability in culture media. *Plant Physiol.* 81 934–936. 10.1104/pp.81.3.934 16664930 PMC1075455

[B22] Dunlap J. R. Robacker K. M. (1988). Nutrient salts promote light-induced degradation of indole-3-acetic Acid in tissue culture media. *Plant Physiol.* 88 379–382. 10.1104/pp.88.2.379 16666312 PMC1055585

[B23] Fu J. Wang S. (2011). Insights into auxin signaling in plant-pathogen interactions. *Front. Plant Sci.* 2:74. 10.3389/fpls.2011.00074 22639609 PMC3355572

[B24] Fu L. Wang M. Han B. Tan D. Sun X. Zhang J. (2016). Arabidopsis myrosinase genes AtTGG4 and AtTGG5 are root-tip specific and contribute to auxin biosynthesis and root-growth regulation. *Int. J. Mol. Sci.* 17:E892. 10.3390/ijms17060892 27338341 PMC4926426

[B25] Geu-Flores F. Møldrup M. E. Bottcher C. Olsen C. E. Scheel D. Halkier B. A. (2011). Cytosolic γ-glutamyl peptidases process glutathione conjugates in the biosynthesis of glucosinolates and camalexin in *Arabidopsis*. *Plant Cell* 23 2456–2469. 10.1105/tpc.111.083998 21712415 PMC3160024

[B26] Gray W. M. Kepinski S. Rouse D. Leyser O. Estelle M. (2001). Auxin regulates SCF(TIR1)-dependent degradation of AUX/IAA proteins. *Nature* 414 271–276. 10.1038/35104500 11713520

[B27] Grob K. Matile P. (1979). Vauolar location of glucosinolates in horseradish root cells. *Plant Sci. Lett.* 14 327–335. 10.1016/S0304-4211(79)90281-5

[B28] Grsic-Rausch S. Kobelt P. Siemens J. M. Bischoff M. Ludwig-Müller J. (2000). Expression and localization of nitrilase during symptom development of the clubroot disease in Arabidopsis. *Plant Physiol.* 122 369–378. 10.1104/pp.122.2.369 10677430 PMC58874

[B29] Halkier B. A. Gershenzon J. (2006). Biology and biochemistry of glucosinolates. *Annu. Rev. Plant Biol.* 57 303–333. 10.1146/annurev.arplant.57.032905.105228 16669764

[B30] Hayashi K. Neve J. Hirose M. Kuboki A. Shimada Y. Kepinski S. (2012). Rational design of an auxin antagonist of the SCF(TIR1) auxin receptor complex. *ACS Chem. Biol.* 7 590–598. 10.1021/cb200404c 22234040

[B31] Hayashi K.-I. Tan X. Zheng N. Hatate T. Kimura Y. Kepinski S. (2008). Small-molecule agonists and antagonists of F-box protein-substrate interactions in auxin perception and signaling. *Proc. Natl. Acad. Sci. U.S.A.* 105 5632–5637. 10.1073/pnas.0711146105 18391211 PMC2291130

[B32] Hirsch-Hoffmann M. Gruissem W. Baerenfaller K. (2012). Pep2Pro: the high-throughput proteomics data processing, analysis, and visualization tool. *Front. Plant Sci.* 3:123. 10.3389/fpls.2012.00123 22701464 PMC3371593

[B33] Hrncirik K. Valusek J. Velisek J. (1998). A study on the formation and stability of ascorbigen in an aqueous system. *Food Chem.* 63 349–355. 10.1016/S0308-8146(98)00016-8

[B34] Huot B. Yao J. Montgomery B. L. He S. Y. (2014). Growth-defense tradeoffs in plants: a balancing act to optimize fitness. *Mol. Plant* 7 1267–1287. 10.1093/mp/ssu049 24777989 PMC4168297

[B35] Janowitz T. Trompetter I. Piotrowski M. (2009). Evolution of nitrilases in glucosinolate-containing plants. *Phytochemistry* 70 1680–1686. 10.1016/j.phytochem.2009.07.028 19698961

[B36] Katz E. Nisani S. Sela M. Behar H. Chamovitz D. A. (2015a). The effect of indole-3-carbinol on PIN1 and PIN2 in Arabidopsis roots. *Plant Signal. Behav.* 10:e1062200. 10.1080/15592324.2015.1062200 26252364 PMC4883967

[B37] Katz E. Nisani S. Yadav B. S. Woldemariam M. G. Shai B. Obolski U. (2015b). The glucosinolate breakdown product indole-3-carbinol acts as an auxin antagonist in roots of *Arabidopsis thaliana*. *Plant J.* 82 547–555. 10.1111/tpj.12824 25758811

[B38] Kim J. H. Lee B. W. Schroeder F. C. Jander G. (2008). Identification of indole glucosinolate breakdown products with antifeedant effects on *Myzus persicae* (green peach aphid). *Plant J.* 54 1015–1026. 10.1111/j.1365-313X.2008.03476.x 18346197

[B39] Klein A. P. Sattely E. S. (2017). Biosynthesis of cabbage phytoalexins from indole glucosinolate. *Proc. Natl. Acad. Sci. U.S.A.* 114 1910–1915. 10.1073/pnas.1615625114 28154137 PMC5338394

[B40] Koroleva O. A. Gibson T. M. Cramer R. Stain C. (2010). Glucosinolate-accumulating S-cells in Arabidopsis leaves and flower stalks undergo programmed cell death at early stages of differentiation. *Plant J.* 64 456–469. 10.1111/j.1365-313X.2010.04339.x 20815819

[B41] Kutz A. Müller A. Hennig P. Kaiser W. M. Piotrowski M. Weiler E. W. (2002). A role for nitrilase 3 in the regulation of root morphology in sulphur-starving Arabidopsis thaliana. *Plant J.* 30 95–106. 10.1046/j.1365-313X.2002.01271.x 11967096

[B42] Lehmann T. Janowitz T. Sánchez-Parra B. Alonso M.-M. P. Trompetter I. Piotrowski M. (2017). Arabidopsis NITRILASE 1 contributes to the regulation of root growth and development through modulation of auxin biosynthesis in seedlings. *Front. Plant Sci.* 8:36. 10.3389/fpls.2017.00036 28174581 PMC5258727

[B43] Ludwig-Müller J. Pieper K. Ruppel M. Cohen J. D. Epstein E. Kiddle G. (1999). Indole glucosinolate and auxin biosynthesis in *Arabidopsis thaliana* (L.) Heynh. glucosinolate mutants and the development of clubroot disease. *Planta* 208 409–419. 10.1007/s004250050576 10384731

[B44] Ludwig-Müller J. Prinsen E. Rolfe S. A. Scholes J. D. (2009). Metabolism and plant hormone action during clubroot disease. *J. Plant Growth Regul.* 28 229–244. 10.1007/s00344-009-9089-4

[B45] Ludwig-Müller J. Schuller A. (2008). What can we learn from clubroots: alterations in host roots and hormone homeostasis caused by *Plasmodiophora brassicae*. *Eur. J. Plant Pathol.* 121 291–302. 10.1007/s10658-007-9237-2

[B46] Lüthy B. Matile P. (1984). The mustard oil bomb: rectified analysis of the subcellular organisation of the myrosinase system. *Biochem. Physiol. Pflanzen* 179 5–12. 10.1016/S0015-3796(84)80059-1

[B47] Maag D. Köhler A. Robert C. A. M. Frey M. Wolfender J. Turlings T. C. J. Angela K. (2016). Highly localized and persistent induction of Bx1 -dependent herbivore resistance factors in maize. *Plant J.* 88 976–991. 10.1111/tpj.13308 27538820

[B48] Machado R. A. Robert C. A. Arce C. C. Ferrieri A. P. Xu S. Jimenez-Aleman G. H. (2016). Auxin is rapidly induced by herbivore attack and regulates a subset of systemic, jasmonate-dependent defenses. *Plant Physiol.* 172 521–532. 10.1104/pp.16.00940 27485882 PMC5074610

[B49] Madsen S. R. Olsen C. E. Nour-Eldin H. H. Halkier B. A. (2014). Elucidating the role of transport processes in leaf glucosinolate distribution. *Plant Physiol.* 166 1450–1462. 10.1104/pp.114.246249 25209984 PMC4226354

[B50] Mangan S. Alon U. (2003). Structure and function of the feed-forward loop network motif. *Proc. Natl. Acad. Sci. U.S.A.* 100 11980–11985. 10.1073/pnas.2133841100 14530388 PMC218699

[B51] McDanell R. McLean A. E. Hanley A. B. Heaney R. K. Fenwick G. R. (1987). Differential induction of mixed-function oxidase (MFO) activity in rat liver and intestine by diets containing processed cabbage: correlation with cabbage levels of glucosinolates and glucosinolate hydrolysis products. *Food Chem. Toxicol.* 25 363–368. 10.1016/0278-6915(87)90170-0 3609976

[B52] Nakano R. T. Piślewska-Bednarek M. Yamada K. Edger P. P. Miyahara M. Kondo M. (2017). PYK10 myrosinase reveals a functional coordination between er bodies and glucosinolates in *Arabidopsis thaliana*. *Plant J.* 204–220. 10.1111/tpj.13377 27612205

[B53] Nakano R. T. Yamada K. Bednarek P. Nishimura M. Hara-Nishimura I. (2014). ER bodies in plants of the *Brassicales* order: biogenesis and association with innate immunity. *Front. Plant Sci.* 5:73. 10.3389/fpls.2014.00073 24653729 PMC3947992

[B54] Naseem M. Kaltdorf M. Dandekar T. (2015). The nexus between growth and defence signalling: auxin and cytokinin modulate plant immune response pathways. *J. Exp. Bot.* 66 4885–4896. 10.1093/jxb/erv297 26109575

[B55] Nitz I. Berkefeld H. Puzio P. S. Grundler F. M. W. (2001). Pyk10, a seedling and root specific gene and promoter from *Arabidopsis thaliana*. *Plant Sci.* 161 337–346. 10.1016/S0168-9452(01)00412-5 11448764

[B56] Normanly J. Grisafi P. Fink G. R. Bartel B. (1997). Arabidopsis mutants resistant to the auxin effects of indole-3-acetonitrile are defective in the nitrilase encoded by the NIT1 gene. *Plant Cell* 9 1781–1790. 10.1105/tpc.9.10.1781 9368415 PMC157021

[B57] Piekarska A. Kusznierewicz B. Meller M. Dziedziul K. Namieśnik J. Bartoszek A. (2013). Myrosinase activity in different plant samples; optimisation of measurement conditions for spectrophotometric and pH-stat methods. *Ind. Crops Prod.* 50 58–67. 10.1016/j.indcrop.2013.06.048

[B58] Piotrowski M. Scho S. Weiler E. W. Nit A. (2001). The *Arabidopsis thaliana* isogene NIT4 and its orthologs in tobacco encode β-Cyano- L -alanine Hydratase / Nitrilase. *J. Biol. Chem.* 276 2616–2621. 10.1074/jbc.M007890200 11060302

[B59] Preobrazhenskaya M. N. Bukhman V. M. Korolev A. M. Efimov S. A. (1993). Ascorbigen and other indole-derived compounds from Brassica vegetables and their analogs as anticarcinogenic and immunomodulating agents. *Pharmacol. Ther.* 60 301–313. 10.1016/0163-7258(93)90012-3 8022861

[B60] Robert-Seilaniantz A. Grant M. Jones J. D. (2011). Hormone crosstalk in plant disease and defense: more than just jasmonate-salicylate antagonism. *Annu. Rev. Phytopathol.* 49 317–343. 10.1146/annurev-phyto-073009-114447 21663438

[B61] Schlaeppi K. Abou-mansour E. Buchala A. Mauch F. (2010). Disease resistance of Arabidopsis to *Phytophthora brassicae* is established by the sequential action of indole glucosinolates and camalexin. *Plant J.* 62 840–851. 10.1111/j.1365-313X.2010.04197.x 20230487

[B62] Semsey S. (2014). A mixed incoherent feed-forward loop allows conditional regulation of response dynamics. *PLoS One* 9:e91243. 10.1371/journal.pone.0091243 24621982 PMC3951346

[B63] Staub R. E. Feng C. Onisko B. Bailey G. S. Firestone G. L. Bjeldanes L. F. (2002). Fate of indole-3-carbinol in cultured human breast tumor cells. *Chem. Res. Toxicol.* 15 101–109. 10.1021/tx010056m 11849035

[B64] Strader L. C. Zhao Y. (2016). Auxin perception and downstream events. *Curr. Opin. Plant Biol.* 33 8–14. 10.1016/j.pbi.2016.04.004 27131035 PMC5050066

[B65] Tan X. Calderon-Villalobos L. I. Sharon M. Zheng C. Robinson C. V. Estelle M. (2007). Mechanism of auxin perception by the TIR1 ubiquitin ligase. *Nature* 446 640–645. 10.1038/nature05731 17410169

[B66] Trott O. Olson A. J. (2010). AutoDock Vina. *J. Comput. Chem.* 31 445–461. 10.1002/jcc.21334 PMC3041641 19499576

[B67] Tyson J. J. Novák B. (2010). Functional motifs in biochemical reaction networks. *Annu. Rev. Phys. Chem.* 61 219–240. 10.1146/annurev.physchem.012809.103457 20055671 PMC3773234

[B68] Vorwerk S. Biernacki S. Hillebrand H. Janzik I. Müller A. Weiler E. W. (2001). Enzymatic characterization of the recombinant *Arabidopsis thaliana* nitrilase subfamily encoded by the *NIT2/NIT1/NIT3*-gene cluster. *Planta* 212 508–516. 10.1007/s004250000420 11525507

[B69] Wang D. Pajerowska-Mukhtar K. Culler A. H. Dong X. (2007). Salicylic acid inhibits pathogen growth in plants through repression of the auxin signaling pathway. *Curr. Biol.* 17 1784–1790. 10.1016/j.cub.2007.09.025 17919906

[B70] Wentzell A. M. Kliebenstein D. J. (2008). Genotype, age, tissue, and environment regulate the structural outcome of glucosinolate activation. *Plant Physiol.* 147 415–428. 10.1104/pp.107.115279 18359845 PMC2330308

[B71] Wienkoop S. Weiss J. May P. Kempa S. Irgang S. Recuenco-Munoz L. (2010). Targeted proteomics for *Chlamydomonas reinhardtii* combined with rapid subcellular protein fractionation, metabolomics and metabolic flux analyses. *Mol. Biosyst.* 6 1018–1031. 10.1039/b920913a 20358043

[B72] Wittstock U. Burow M. (2010). Glucosinolate breakdown in Arabidopsis: mechanism, regulation and biological significance. *Arabidopsis Book* 8:e0134. 10.1199/tab.0134 22303260 PMC3244901

[B73] Wittstock U. Kliebenstein D. J. Lambrix V. Reichelt M. Gershenzon J. (2003). “Glucosinolate hydrolysis and its impact on generalist and specialist insect herbivores,” in *Integrative Phytochemistry: From Ethnobotany to Molecular Ecology* Vol. 37 ed. Romeo J. T. (Amsterdam: Elsevier) 101–125.

[B74] Wittstock U. Meier K. Dörr F. Ravindran B. M. (2016). NSP-dependent simple nitrile formation dominates upon breakdown of major aliphatic glucosinolates in roots, seeds, and seedlings of *Arabidopsis thaliana* columbia-0. *Front. Plant Sci.* 7:1821. 10.3389/fpls.2016.01821 27990154 PMC5131009

[B75] Yamada T. (1993). The role of auxin in plant-disease development. *Annu. Rev. Phytopathol.* 31 253–273. 10.1146/annurev.py.31.090193.001345 18643769

[B76] Zhang H. He H. Wang X. Wang X. Yang X. Li L. (2011). Genome-wide mapping of the *HY5*-mediated genenetworks in Arabidopsis that involve both transcriptional and post-transcriptional regulation. *Plant J.* 65 346–358. 10.1111/j.1365-313X.2010.04426.x 21265889

[B77] Zhao Y. Wang J. Liu Y. Miao H. Cai C. Shao Z. (2015). Classic myrosinase-dependent degradation of indole glucosinolate attenuates fumonisin B1-induced programmed cell death in Arabidopsis. *Plant J.* 81 920–933. 10.1111/tpj.12778 25645692

