# Supplementary figures and images for: Dynamic Modeling of Indole Glucosinolate Hydrolysis and Its Impact on Auxin Signaling
Source: Front Plant Sci. 2018 Apr 26;9:550. doi: 10.3389/fpls.2018.00550 (PMC5932361; doi:10.3389/fpls.2018.00550)

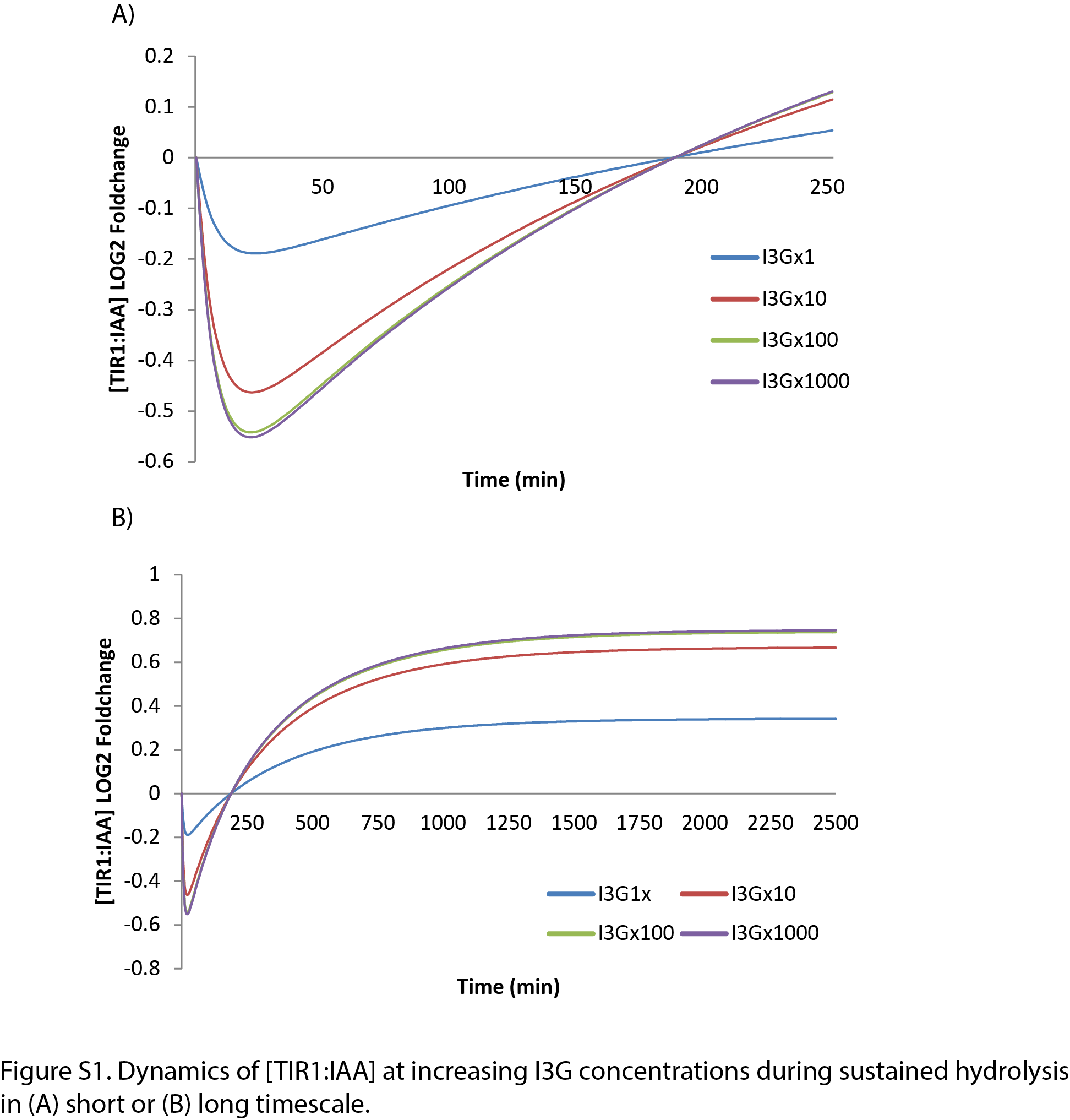

Supplement: Supplementary file 1 [file Image_1.TIF]

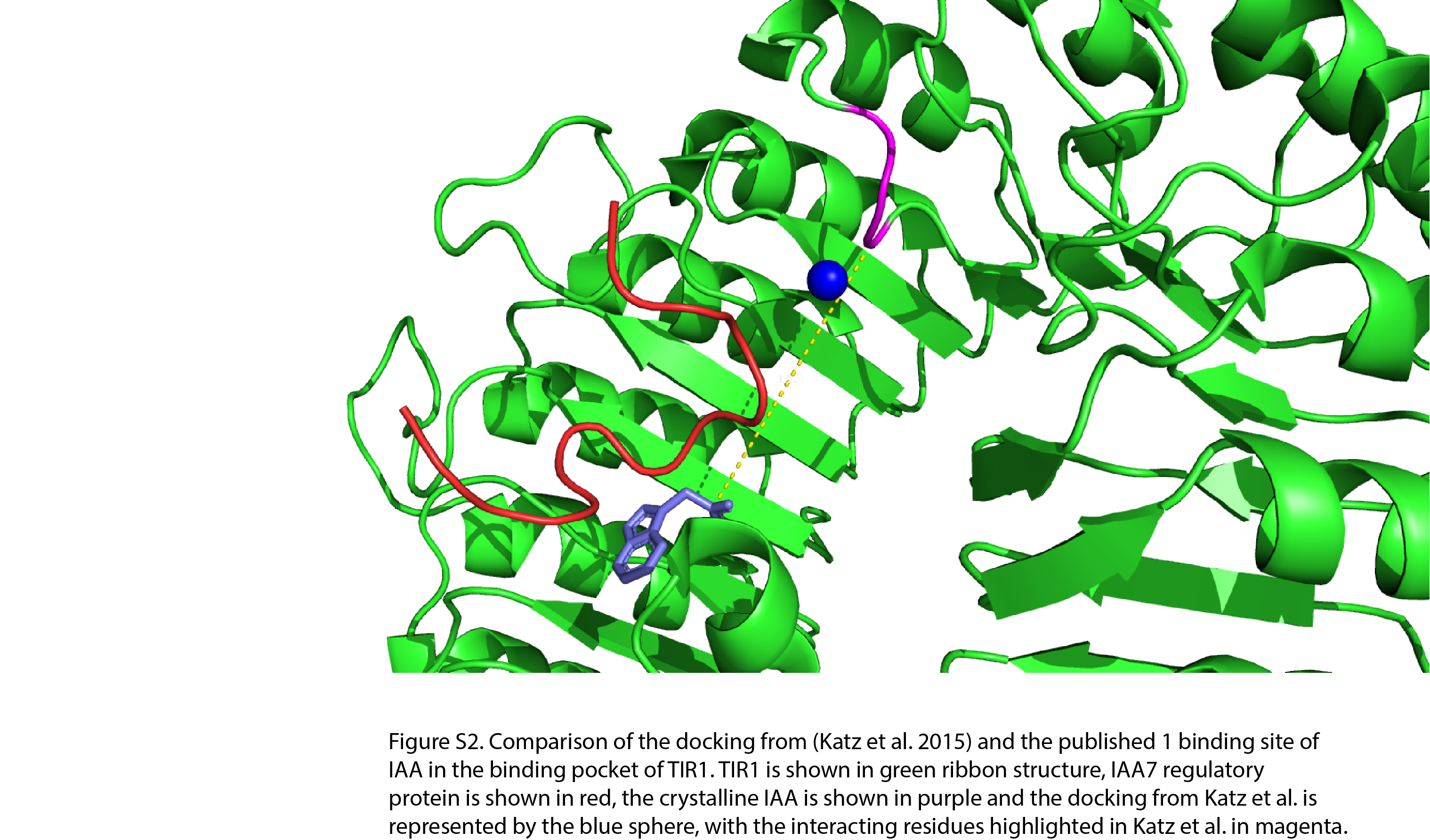

Supplement: Supplementary file 2 [file Image_2.TIF]
